# Baseline-dependent effects of amphetamine on attention are associated with striatal dopamine metabolism

**DOI:** 10.1038/s41598-017-00437-9

**Published:** 2017-03-22

**Authors:** Karly M. Turner, James Peak, Thomas H. J. Burne

**Affiliations:** 1 Queensland Brain Institute, The University of Queensland, St Lucia, QLD 4072 Australia; 2 Queensland Centre for Mental Health Research, The Park Centre for Mental Health, Richlands, QLD 4077 Australia

## Abstract

Psychostimulants, such as amphetamine, are widely used to treat attentional deficits. In humans, response to dopaminergic medications is complex with improvement often dependent on baseline performance. Our goal was to determine if attention in rats could be improved by low dose amphetamine in a baseline-dependent manner by examining the relationship between task performance, drug response and monoamine levels in corticostriatal tissue. Firstly, rats performed a signal detection task with varying signal durations before administration of saline, 0.1 or 0.25 mg/kg amphetamine. Following 0.1 mg/kg amphetamine, accuracy in poor performing individuals increased to that of high performing rats. Furthermore, baseline accuracy correlated with the magnitude of improvement after amphetamine. Secondly, neurochemical analysis of monoamine content and gene expression levels in the prefrontal cortex (PFC) and dorsal striatum (CPU) was conducted. CPU homovanillic acid and 5-hydroxyindoleacetic acid levels were increased in poor performers with a significant correlation between the expression of the dopamine transporter gene and baseline accuracy. No changes were found in the PFC. These results indicated poor performance was associated with greater response to amphetamine and altered DA and 5-HT neurotransmitter systems in CPU. These results suggest striatal monoamine function may be fundamental to explaining individual differences in psychostimulant response.

## Introduction

Altered catecholamine functioning in cortical and striatal brain regions have been implicated in a number of neuropsychiatric disorders, such as schizophrenia, Parkinson’s disease and attention deficit hyperactivity disorder (ADHD). Cognitive deficits are a prominent feature of these disorders and drugs acting on catecholamine pathways are often used for treatment. Therefore, it is critical to investigate the complex relationship between catecholamine activity in the brain, cognitive functioning and the effect of medications.

Psychostimulants are highly effective in the treatment of attentional deficits in ADHD, but are increasingly being used for performance enhancement in healthy individuals^1–3^. Irrespective of diagnosis, psychostimulants can improve performance in individuals with low baseline performance and an individual’s response has been linked to a range of neurological changes^4, 5^. Studies have focused on the role of cortical and striatal brain regions, which are the likely site of neurobiological deficits and medication targets^4, 6–8^. However, response to psychostimulants is complex with positive and negative changes in cognitive performance dependent on dose and task; as well as individual characteristics such as baseline performance, genetics and state when tested (e.g. stress, tiredness)^9^. The attention-improving effects of psychostimulants, such as methylphenidate and amphetamine, have been examined in animal models. Methylphenidate has been shown to improve performance on a range of tests, both at the group level and in poor-performing animals^10–14^. However, despite being one of the mainstream treatment for improving attentional deficits in humans, low doses of amphetamine have only improved attention in a handful of preclinical studies^12, 15–17^. This may be due to species-specific differences in drug metabolism or because the relationship between cognitive performance and catecholamine function is very complex.

Studies in humans have demonstrated the importance of considering individual differences in response to pharmacological treatment^6^. For example, improvement after amphetamine has been shown to be dependent on baseline performance, such that low performers have the greatest improvement^4, 18^, which has also been found for methylphenidate^5, 19^. However, rodent studies commonly compare groups of subjects, rather than taking advantage of the variability between individuals^20, 21^. To enhance individual variability, it should be advantageous to use an outbred strain, such as Sprague-Dawley rats. Individual variability in Sprague-Dawley rats has been used to demonstrate the relationship between working memory performance and response to L-745,870, a selective D4 antagonist in rats^22^; as well as between working memory performance and locomotor response to amphetamine^20^. These findings support the hypothesis that individual differences in baseline functioning and response to dopaminergic agents are linked.

The aim of this study was to determine if attentional performance in rats could be improved with low dose amphetamine, and to examine the relationship between task performance, drug response and catecholamine levels in corticostriatal tissue. Firstly, it was hypothesised that low dose amphetamine would improve accuracy on a signal detection task in low performing rats. Secondly, it was expected that the improvement in performance would correlate negatively with baseline accuracy. Finally, this study examined differences in post-mortem catecholamine levels and gene expression within two brain regions implicated in cognition; the dorsal striatum and prefrontal cortex. Our results suggest that rodents can be used to investigate amphetamine-induced improvement in attention and that striatal dopaminergic and serotonergic systems are associated with differences in attentional performance.

## Results

**Figure 1:**
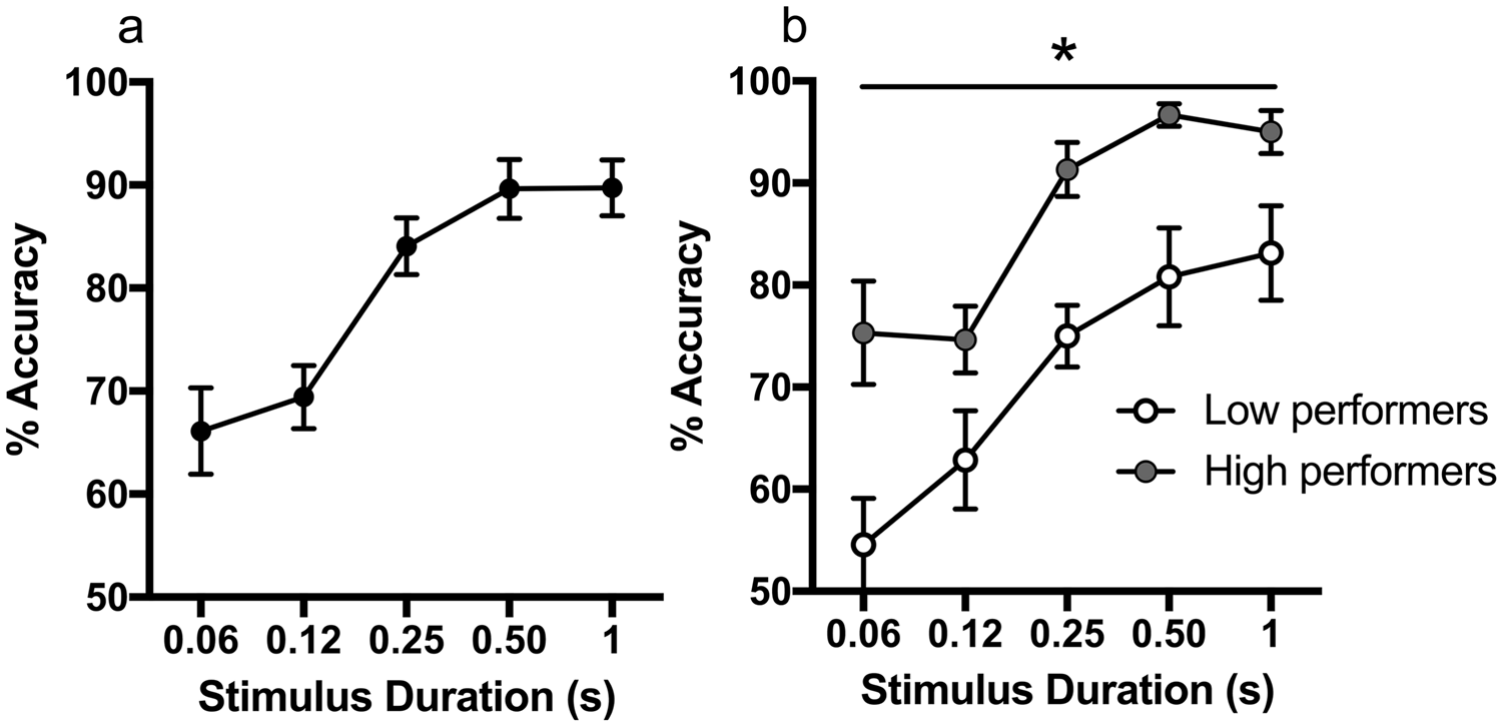
Manipulating accuracy by reducing signal duration. (**a**) Decreasing signal duration led to a reduction in accuracy on the signal detection task where 50% is chance accuracy (*N* = 18). (**b**) There was a significant difference between low and high performance groups (*n* = 8–10) across all signal durations, *p < 0.05.

Rats were initially trained on the Signal Detection Task and tested using reducing signal durations^23^. The task requires rats to pay attention to a stimulus panel and respond either left or right depending on whether they see the panel illuminate or the lights remain off. To make the task more difficult, the signal was presented at reduced durations (0.06, 0.12, 0.25, 0.5 or 1 s). Accuracy in response to 0.5 and 1.0 s stimuli was very high (90%) and appeared to plateau due to ceiling effects, but was reduced with brief signal durations (Fig. 1a). Using a within-animal design, rats were then treated with vehicle, 0.1 or 0.25 mg/kg amphetamine prior to testing. Previously, it was found that the procognitive effects of low dose amphetamine were dependent on baseline performance and therefore drug response was compared between low and high performance groups^24^. A power analysis using these previous results was calculated to determine the number of animals required for the current study. A median split (at 80% accuracy) from baseline performance was used to allocate rats into low (*n* = 8) and high (*n* = 10) performance groups. Repeated measures ANOVA was used to compare groups across signal durations at baseline and after amphetamine. Following this significant differences between performance groups were assessed using two-tailed independent *t*-tests and paired *t*-test where required. Bonferroni adjustment for multiple comparisons was used where appropriate.

### Pharmacology

**Figure 2:**
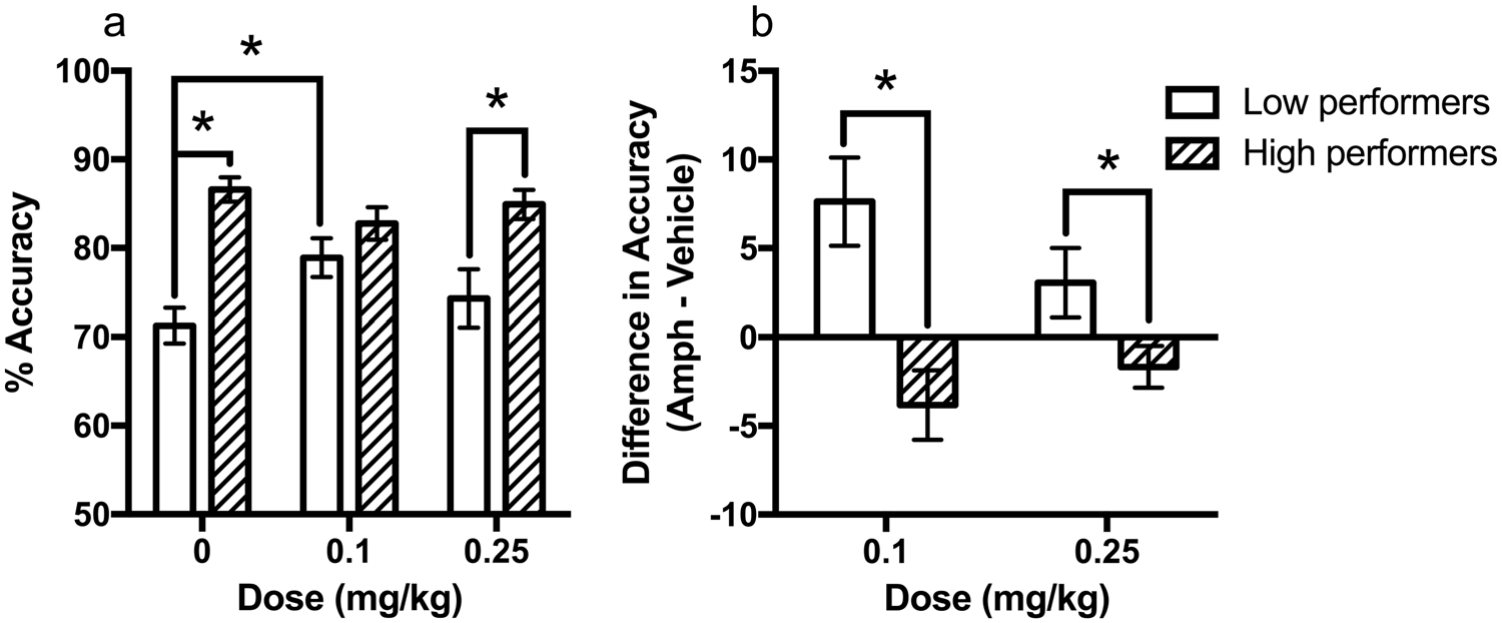
Performance accuracy after amphetamine. (**a**) The reduced accuracy observed in the low performing group after vehicle was corrected by 0.1 mg/kg amphetamine, but not by 0.25 mg/kg amphetamine. (**b**) A difference score was calculated to compare accuracy after vehicle and amphetamine, demonstrating a significant difference between low and high performing rats in response to 0.1 mg/kg amphetamine and 0.25 mg/kg amphetamine (*n* = 8–10), *p < 0.05.

There was a main effect of Duration after vehicle treatment (*F*_(3.6,64)_ = 18.59, *p* < 0.001) and a significant difference in accuracy between low and high performers across all durations as expected (*F*_(1,16)_ = 42.51, *p* < 0.001, Fig. 1b). A repeated measures ANOVA with three drug levels (Dose: vehicle, 0.1, 0.25 mg/kg), five signal durations (Duration: 0.06 s, 0.12 s, 0.25, 0.5, 1 s) and a between subjects factor of performance group (Group) found a main effect of Duration (*F*_(4,64)_ = 51.99, *p* < 0.001), Group (*F*_(1,16)_ = 17.21, *p* = 0.001) and a Dose x Group interaction (*F*_(2,32)_ = 7.98, *p* = 0.002). These results indicated that overall accuracy was reduced with shorter signal duration across all drug doses. As there was no interaction between Group and Duration or Dose and Duration, accuracy across the five signal durations was averaged for each individual. Using the average accuracy across durations, independent *t*-tests revealed that Groups differed after vehicle (*t*_(16)_ = −6.52, *p* < 0.001), were not different after 0.1 mg/kg (*t*_(16)_ = −1.35, *p* = 0.195, Fig. 2a) but were again different after 0.25 mg/kg amphetamine (*t*_(16)_ = −3.08, *p* = 0.007, Fig. 2a). Paired samples *t*-tests found that the low performing group significantly improved after 0.1 mg/kg (*t*_(7)_ = −3.07, *p* = 0.018) but not 0.25 mg/kg amphetamine (*t*_(9)_ = −1.56, *p* = 0.163). However, the high performing group did not have a significant change in accuracy after either dose. When compared using a difference score (accuracy after amphetamine – accuracy after vehicle) the groups varied in response to 0.1 mg/kg amphetamine (*t*_(16)_ = 3.67, *p* = 0.002, Fig. 2b) and 0.25 mg/kg amphetamine (*t*_(16)_ = 2.16, *p* = 0.046, Fig. 2b), suggesting that compared to baseline accuracy the low performing group did better after amphetamine but the high performing group did not. The key result from these findings was that the low performing group, but not the high performing group, improved in accuracy after 0.1 mg/kg amphetamine.

**Figure 3:**
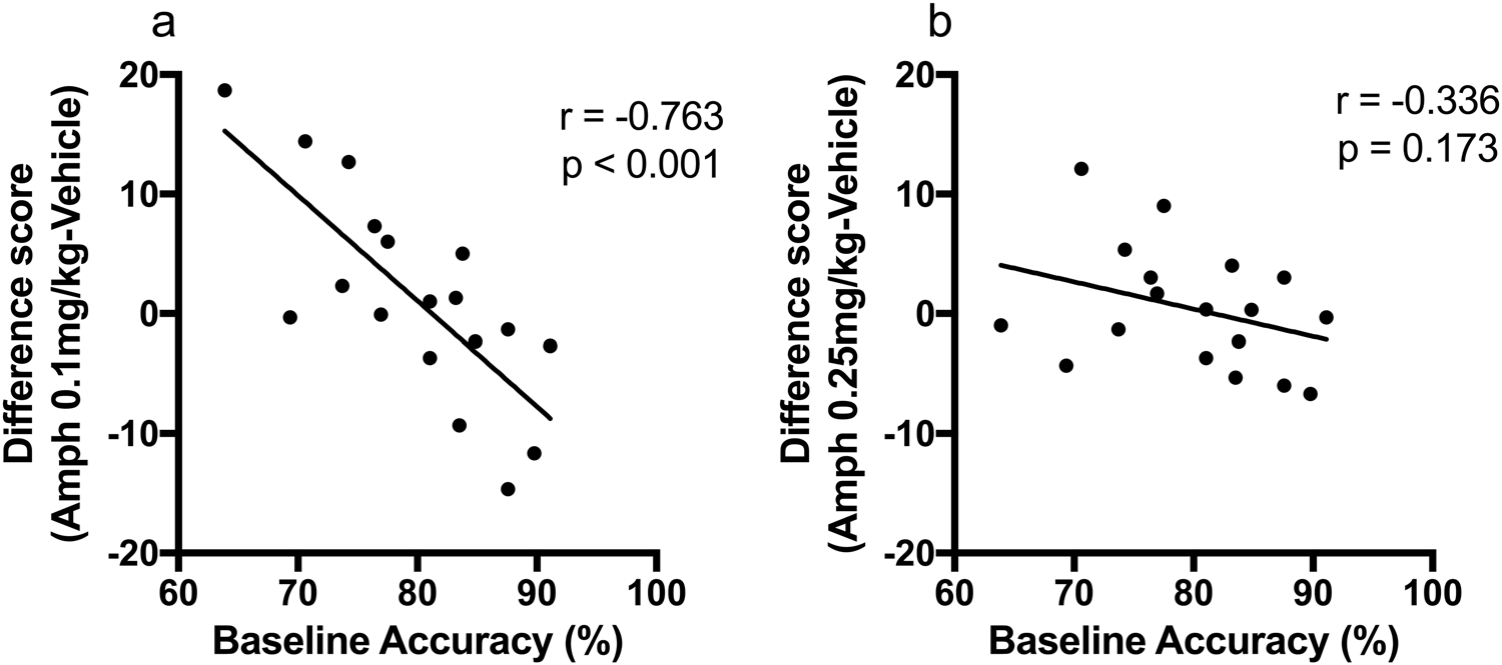
After adjustment for regression to the mean effects response to amphetamine was found to negatively correlate with baseline performance after (**a**) 0.1 mg/kg amphetamine (*r* = −0.763, *p* < 0.001) but not (**b**) 0.25 mg/kg amphetamine (*r* = −0.336, *p* = 0.173), (*N* = 18).

To determine if there was a continuous relationship between baseline performance and response to amphetamine, a correlational analysis was conducted using the whole cohort of rats (N = 18). A significant negative correlation was observed for 0.1 mg/kg (*r* = −0.764, *p* < 0.001) but not 0.25 mg/kg amphetamine (*r* = −0.336, *p* = 0.173). Because the relationship was negative, scores were adjusted for regression to the mean effects. The adjusted baseline score was also significantly correlated with the recalculated difference score after 0.1 mg/kg (*r* = −0.763, *p* < 0.001, Fig. 3a) but not after 0.25 mg/kg amphetamine.

There were no significant effects of Group or Dose with respect to other measures including non-signal accuracy, response latency, latency to initiate trials, or the number of additional head entries made during the session. This is important because it indicates that the performance groups did not differ on other aspects of task performance, such as motivation. It also indicates that the doses of amphetamine used did not induce a locomotor phenotype or reduce task engagement. At the conclusion of the drug schedule, all rats were treated with vehicle and tested again. There was a significant difference in accuracy between Groups (*t*_(16)_ = −2.71, *p* = 0.015) on the final day and a strong correlation between initial and final testing days (*r* = 0.817, *p* < 0.001), demonstrating stability of the performance measure and the reversible effect of acute amphetamine administration.

### Neurochemistry

**Figure 4:**
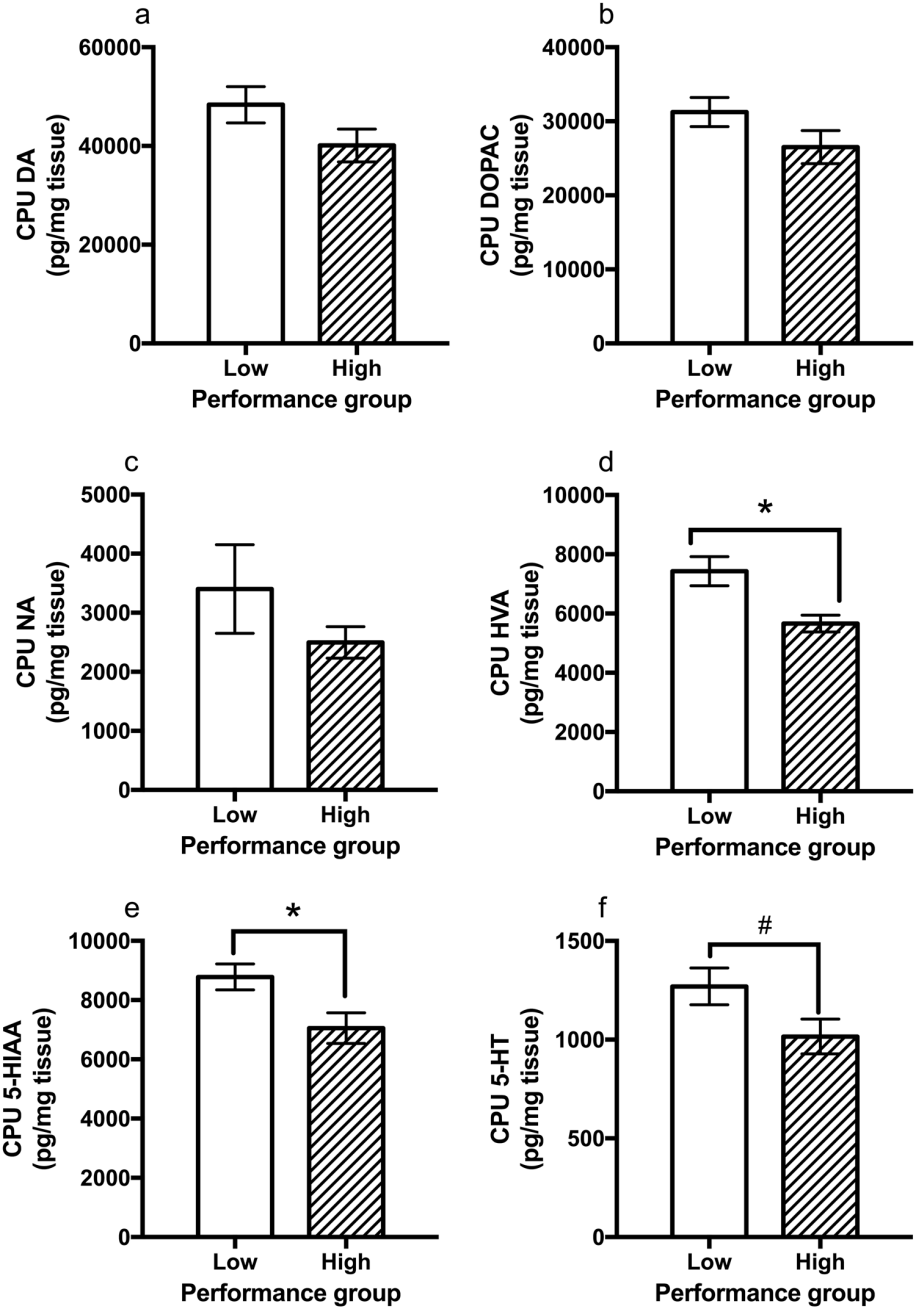
Catecholamine levels in the striatum (CPU) of low and high performing rats. Performance groups did not significantly differ in (**a**) dopamine (DA), (**b**) dihydroxyphenyl acetic acid (DOPAC) or (**c**) noradrenaline (NA). However, low performing rats had significantly more (**d**) homovanillic acid (HVA) and (**e**) 5-hydroxyindoleacetic acid (5-HIAA) than high performing rats with (**f**) serotonin (5-HT) showing a non-significant trend in the same direction. ^#^ *p* = 0.065, *p < 0.05.

**Figure 5:**
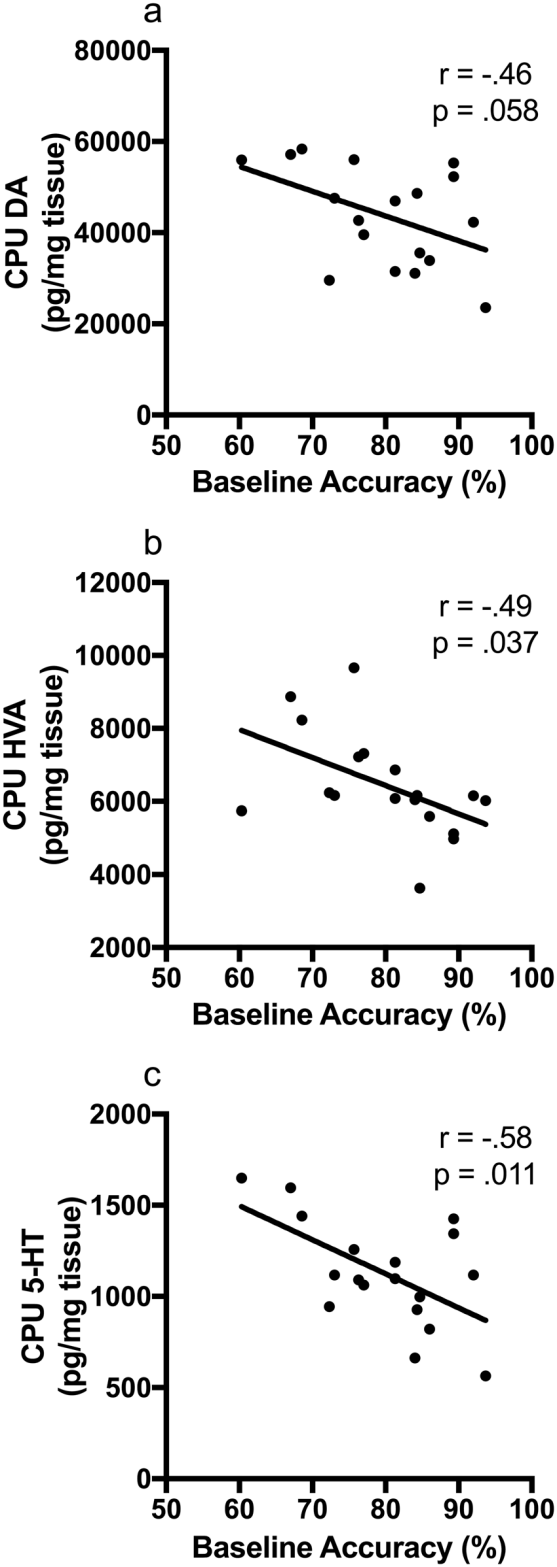
Striatal (CPU) levels of (**a**) dopamine (DA) showed a negative trend but were not significantly correlated with baseline performance accuracy (*r* = −0.46, *p* = 0.058). However, (**b**) homovanillic acid (HVA) (*r* = −0.49, *p* = 0.037) and (**c**) serotonin (5-HT) levels (*r* = −0.58, *p* = 0.011) were significantly negatively correlated with baseline performance (*N* = 18).

At the completion of testing, the striatum (CPU) and medial prefrontal cortex (PFC) were dissected for neurochemical analysis using high performance liquid chromatography (HPLC). Low performing rats had significantly higher striatal HVA levels than high performing rats (*t*_(16)_ = 3.26, *p* = 0.005, Fig. 4d) with a subsequent increase in the ratio of HVA to 3MT (*t*_(16)_ = 2.14, *p* = 0.048). Furthermore, a significant negative correlation was found across the cohort between baseline accuracy and striatal HVA levels (*r* = −0.494, *p* = 0.037, *N* = 18, Fig. 5b) and a negative non-significant relationship was found for striatal DA levels (*r* = −0.455, *p* = 0.058, *N* = 18, Fig. 5a). In addition, there was a significant increase in 5-HIAA levels in low performing rats (*t*_(16)_ = 2.48, *p* = 0.025, *N* = 18, Fig. 4e). Although failing to reach significance, a similar pattern between groups was found for 5-HT (*t*_(16)_ = 1.98, *p* = 0.065, *N* = 18, Fig. 4f), which lead to a significant negative correlation with baseline accuracy (*r* = −0.584, *p* = 0.011, *N* = 18, Fig. 5c). There were no significant effects of Group on other monoamine levels in the CPU (Fig. 4a,b,c) or PFC (Supplementary Fig. 1), and there were no significant differences between low and high performing rats in the ratios of DOPAC/DA, HVA/DA or 5-HIAA/5-HT in either the PFC or CPU (Supplementary Table 1). However, there were many biologically meaningful significant correlations between neurochemical measures in the CPU, such as associations between HVA and DOPAC (*r* = −0.735, *p* = 0.001, *N* = 18) or HVA and NA (*r* = −0.574, *p* = 0.013, *N* = 18), demonstrating the reliability of HPLC measures.

### PCR

**Figure 6:**
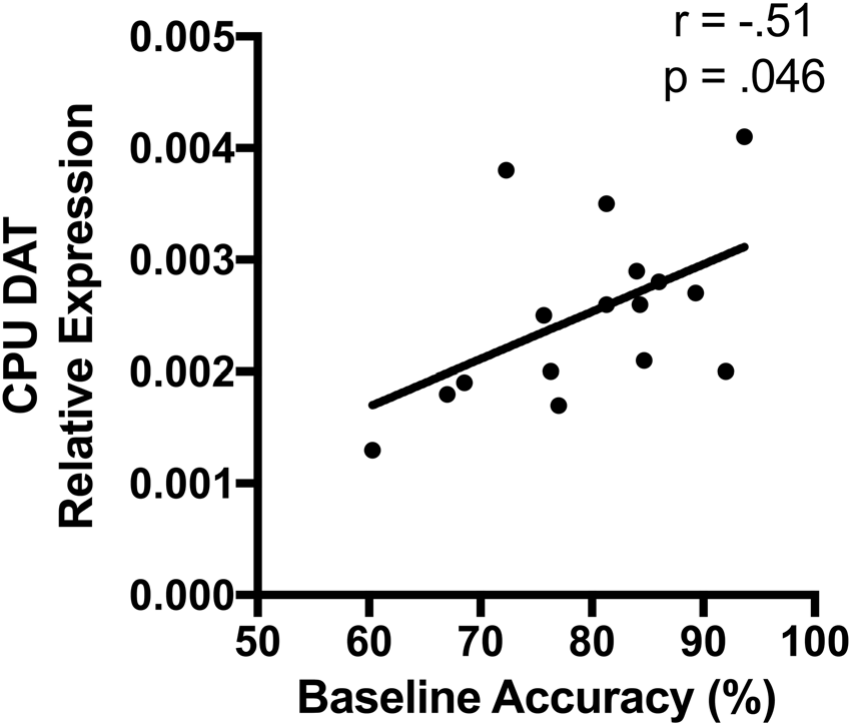
Dopamine transporter (DAT) gene expression in the striatum (CPU) was significantly correlated with baseline task performance such that low performing rats had reduced expression (*r* = 0.506, *p* = 0.046, *N* = 18).

Real-time PCR was conducted on CPU tissue to further investigate changes in dopamine production and metabolism. The aim was to determine if there was a relationship between task performance and levels of tyrosine hydroxylase (TH), dopamine transporter (DAT) and catechol-O-methyl transferase (COMT). It was found that DAT gene expression was positively correlated with baseline task performance (*r* = 0.506, *p* = 0.046, *N* = 18, Fig. 6). There was no significant relationship between performance and expression levels of TH or COMT.

## Discussion

The major findings from this study were that (1) attention was improved in low performing animals using a low dose of amphetamine; (2) baseline performance correlated with the magnitude of improvement after amphetamine; and (3) HVA and 5-HIAA levels were higher in low performing rats, while expression of DAT was reduced in the striatum and the levels correlated with baseline performance. Few studies have shown baseline-dependent effects of amphetamine on attention in rats as demonstrated in this study. Furthermore, we found that individual differences in baseline performance were related to DA and 5-HT systems in the striatum, but not in the prefrontal cortex. These findings are important as they demonstrate that individual differences in attention and response to amphetamine, as well as the monoaminergic mechanisms underpinning this relationship, can be modelled in rats.

Although previous studies using low dose amphetamine in rodents have produced mixed results^11, 15, 17, 25–29^, our findings support the procognitive effects observed on signal detection tasks at reduced stimulus durations^12^. Steps were taken in both the present study and another study demonstrating procognitive effects by Andrzejewski, *et al*.^12^ to improve stability of performance and reduce injection-related stress. Both studies also used decreasing signal durations that minimised ceiling and floor effects. By manipulating task difficulty, accuracy could be used to assess drug effects on challenging trials where performance could be either improved or further impaired. For example, the high performing group was achieving a mean of 97% as a group on a longer signal duration, but averaged only 87% across all signal durations (0.06, 0.12, 0.25, 0.5, 1 s), thereby leaving room for improvement particularly at brief durations. Studies also varied in terms of the ideal dose of amphetamine, however this is likely to relate to study specific details such as task demands, length of training or strain used. A unique consideration in our study was determining how individual responses differed with respect to their baseline performance.

The investigation of individual differences is imperative given that the effects of psychostimulants have been shown to depend on baseline performance in humans. Typically, poor performing individuals have the greatest improvement after psychostimulants^3, 4, 19^. Therefore, finding the same pattern in the current study supports the validity of using animal models to understand the mechanisms of psychostimulant action on attention. However, few studies have explored whether the effects of amphetamine are related to baseline performance accuracy in rodents. Paterson, *et al*.^30^ examined the effects of amphetamine (0.1–1.0 mg/kg) on 5-choice serial reaction time task (5-CSRTT) performance in rats selected for sub-optimal performance (<75% accuracy) but did not find accuracy was improved. In another study, individual differences in response to methylphenidate were noted on a working memory task, whereby performance was optimised at different doses, but not explored further^31^. One of the few reports investigating individual differences in cognitive performance in rodents found baseline working memory performance was correlated with response to L-745,870, a selective D4 antagonist^22^. Together with our data, these previous studies indicate there is sufficient variation in Sprague-Dawley rats to measure individual differences in cognition and pharmacological response. The need for analysis of individual difference in stimulant response was recently highlighted by Bickel, *et al*.^32^. They reanalysed data from preclinical and clinical studies investigating correlations between individual baseline measures of impulsivity and psychostimulant response. They found a significant relationship in the majority of studies using amphetamine (72%). Although it remains to be seen if the same effects would be observed for attentional measures, the current study provides evidence for the baseline dependence of amphetamine action on attention in rodents. Although there is increasing interest in exploring individual differences, many rodent studies thus far have not consider baseline-dependent drug effects on cognition. In addition to using a procognitive drug with a small effect size, these factors may in part explain the inconsistent results for low dose amphetamine.

We next examined monoamine levels in brain regions relevant to task performance to explore the neurobiological differences between individuals. It was predicted that DA levels in the CPU and PFC would be altered. Although there were no significant changes detected in the PFC, striatal levels of the final DA metabolite homovanillic acid (HVA) and the serotonergic metabolite 5-HIAA were greater in the low performing individuals. Furthermore, it was found that low performing rats had reduced expression of DAT. These results are in agreeance with a previous study in mice with genetic knock-down of DAT showing significantly increased striatal HVA, but not PFC HVA levels^33^. Although further studies would be needed to verify functional alterations, reduced re-uptake via DAT could lead to dopamine lingering in the synapse and increase conversion to HVA. Excessive synaptic dopamine could also lead to stimulation of the presynaptic D2 autoreceptors, which act to reduce the readily releasable pool of dopamine. This could potentially alter the ratio of basal to stimulated dopamine release after administration of amphetamine. The increase in 5HIAA in low performers may be due to greater production or greater metabolism of 5-HT. However, given there was also a significant negative correlation between baseline performance and 5-HT levels, the results suggest increased 5-HT production in poor performing rats. It was shown that lesioning the dorsal raphe nucleus with 5,7-DHT (which reduced CPU 5-HT by 78%) resulted in improved accuracy and increased impulsivity in rats on the 5-CSRTT^34^. Similar effects were found in humans where tryptophan depletion also improved accuracy on the 4-CSRTT variant^35^, while simultaneously increasing impulsive responses. These studies indicate lower levels of 5-HT should be associated with greater performance, consistent with the findings of this study.

These neurochemical findings are in agreement with other studies demonstrating that in children with ADHD, greater behavioural response to amphetamine was correlated with higher levels of HVA in cerebrospinal fluid (CSF)^36^. It has also been shown that higher CSF levels of HVA predict a positive response to amphetamine and methylphenidate treatment in children^36^. In addition, dextroamphetamine treatment reduces HVA levels in the CSF of children with ADHD in close association with the magnitude of clinical improvement in symptoms^37^. Furthermore, it was also found that CSF levels of 5-HIAA were correlated with symptom reduction by treatment with AMPH or MPH^36^. All together these results suggest that response to psychostimulants is associated with the production of HVA and 5-HIAA in children with ADHD and supporting the validity of our rodent model.

There is also likely to be a substantial role of noradrenaline in both the performance of cognitive tasks and the role of psychostimulants, although no differences in noradrenaline were detected in either brain region in this study. Monoamine levels were measured post-mortem after a drug washout period with the aim of measuring both neurotransmitter content and gene expression levels in brain tissue. Having now isolated a brain region of relevance to task performance, future experiments could investigate functional changes using an *in vivo* recording technique, such as microdialysis, during drug and task administration to investigate dynamic changes. The results of this study could also be extended by infusing specific monoamine-altering drugs into the PFC or CPU to further test the hypotheses generated from this study. In addition, amphetamine was administered systemically in this experiment, however a localised infusion could be used to isolate the region of drug action. This study used male Sprague Dawley and it would be valuable to ascertain whether the same pattern of drug response holds true in female subjects (given the potential role of oestrogen in modulating dopamine functions) and other rat strains. Studies in humans have indicated an effect of amphetamine on reaction time, however in this experiment rats were not encouraged to respond as fast as possible. There was a 1 s delay from stimulus onset until a response could be made to prevent ‘guessing’ behaviour in rats. Although reaction times can be an important measure in human tasks in terms of speed/accuracy trade-off costs this task was optimised to train rats for accurate responding rather than speed of responding. There were no motoric effects seen at these low doses of amphetamine, indicating the doses used were sub-threshold for the motoric effects often reported for amphetamine.

Cognitive deficits related to dysfunction of corticostriatal catecholamine activity have been found in a variety of neuropsychiatric disorders including schizophrenia, ADHD, OCD and Parkinson’s disease. However, studies exploring the role of DA in cognitive functioning have produced mixed findings with both increasing and decreasing levels of DA improving or impairing performance on different tasks. Here we demonstrate on a signal detection task that poor attentional performance can be improved by low dose amphetamine in rats. Furthermore, the degree of amphetamine-induced improvement was associated with baseline performance levels across the cohort, which has not previously been reported in rodents. Finally, we were able to extend these findings to show that individual differences in baseline performance correlate with dopaminergic and serotonergic systems in the CPU, but not the PFC. Future studies should utilise animal models to further understand how corticostriatal functions differ in individuals during task performance, after administration of psychostimulants and characterise the dynamic interaction between drug and task-related activation.

## Methods

### Animals and housing

Adult male Sprague Dawley (ARC, Australia) rats (N = 18) were housed in pairs in a room maintained at 21 ± 2 °C and 60% humidity and on a 12-h light/dark cycle (lights on 0600 h) in cages with a high top wire lid, aspen chip bedding, nesting and wood chew (Able Scientific, WA, USA). Prior to training, rats were food restricted to 90% of their free-feeding body weight with free access to water. All procedures were performed with approval from The University of Queensland Animal Ethics Committee, in accordance with the National Health and Medical Research Council of Australia.

### Apparatus

Operant rat chambers were contained in ventilated, sound attenuated boxes and all responding occurred on a single wall. The wall contained a central house light, signal display panel and nose poke port, and a food magazine on either side of the nose poke port. Rats were rewarded with 45 mg grain pellets (Bioserv, Frenchtown, NJ, USA) delivered to the food receptacle, which was equipped with a head entry detector. Prior to testing rats were habituated to the dimly lit testing room for 30 min. The chamber was operated using MED-PC for Windows software and interfacing (Med Associates Inc., St. Albans, VT, USA).

### Protocol

The training and testing conditions for the signal detection task (SDT) used in this study has previously been described in detail, including a comparison with other rodent tasks^24^. Briefly, training commenced with a fixed ratio schedule where every head entry into a food receptacle was rewarded until 50 pellets were delivered from each receptacle or after 20 min. After receiving >80 pellets on 2 days, rats were trained to nose poke an illuminated central aperture. After learning to nose poke to initiate trials, the signal detection task was implemented. In this protocol the trial was started by a nose poke after which a panel of 9 green LEDs (5 mm, 80 mcd) were either illuminated on signal trials or remained off on non-signal trials. Following a 1 s delay, both receptacles illuminated and the rat needed to make a choice between left or right. Each side was paired with either signal or non-signal light presentation such that the rat would receive a reward for the correct choice or a brief time out for the incorrect choice. Each animal experienced the same pairing throughout training, with half the group assigned to each combination. A variable inter-trial interval occurred between reward collection and nose poke initiation (1, 2, 3 s) and the session ended after 100 trials or 30 min.

### Signal Duration

By reducing the signal duration, the task difficulty was varied from near chance performance to very high accuracy (55–95% accuracy). Using this manipulation, performance could be assessed across a performance range where both ceiling and floor effects could be avoided. The stimulus durations used were 0.06, 0.12, 0.25, 0.5 and 1 s with both magazines illuminating 1 s after stimulus onset. The sessions consisted of 120 trials broken into three blocks. During the first 20 trials and last 20 trials of the session the standard signal (1 s) and non-signal (0 s) trials were presented equally. The reduced signal durations occurred in the central block of 80 trials with 20 non-signal (0 s) and 15 trials for each of the reduced signal durations (0.06, 0.12, 0.25, 0.5; total 60 trials). Rats were familiar with responding equally to both receptacle sides and using this adjusted ratio accommodates for the increased erroneous non-signal selection at very low signal durations (*unpublished pilot study*). Rats were trained on the signal duration manipulation for a minimum of 15 sessions to ensure performance had plateaued.

### Pharmacology

All rats were treated with d-amphetamine (0.1 mg/kg and 0.25 mg/kg) or saline according to a Latin-square design balanced for performance prior to drug. d-amphetamine (Sigma-Aldrich) was diluted in 0.9% saline and given i.p. at 1 ml/kg, 20 min prior to operant testing. Prior to pharmacology experiments, rats were habituated to the injection procedure over 13 consecutive days to reduce stress and variability in responding during the drug schedule.

### Neurochemistry

After testing was completed rats were housed without testing for a minimum of 12 weeks before being euthanised with an overdose of Lethabarb (Virbac Pty. Ltd., Australia). Micro-dissection of brain regions was rapidly performed and sections were immediately frozen in liquid nitrogen^38^. Criteria for obtaining brain regions was based on Paxinos and Watson^39^ with the medial prefrontal cortex (PFC) consisting of the PrL and IL regions and the dorsal striatum (CPU) consisting of the region labelled CPu. Neurotransmitter analysis was conducted by high performance liquid chromatography (HPLC) to measure dopamine (DA), dihydroxyphenyl acetic acid (DOPAC), homovanillic acid (HVA), serotonin (5-HT), 5-hydroxyindoleacetic acid (5-HIAA) and noradrenaline (NA) against the internal standard deoxyepinephrine (DE). Tissue was prepared for HPLC analysis by sonication in ice cold 0.1 M perchloric acid containing 50 ng/ml DE before centrifuging samples at 13000 rpm for 5 min at 4 °C. After filtering (0.22 µm, 4 mm), 20 ul of supernatant was loaded for a 10 µl injection on the HPLC. An isocratic pump, degasser and autosampler (Model 1100, Agilent Technologies, Inc., CA) were connected to a Sunfire C18 4.6 mm × 100 mm × 5 um column (Waters Corporation, MA) maintained at 30 °C and followed by a Coulochem III electrochemical detector (ESA Laboratories, Inc., MA, USA). A guard cell (Model 5020) and analytic cell (Model 5014B) were operated at −150 and +300 mV (ESA Laboratories, Inc., MA). The mobile phase consisted of 75 mM monosodium phosphate, 1.4 mM octane sulfonic acid and 1 mM EDTA adjusted to pH 4.13 with phosphoric acid, before adjusting to 12% acetonitrile. Flow rate was 1 ml/min with a run time of 10 minutes. Analyte concentrations were determined by calculating peak area relative to internal standard and a standard curve using ChemStation software (Agilent Technologies, Inc., CA).

### Real-time PCR

Rat CPU ribonucleic acid (RNA) was extracted using RNeasy micro kit from Qiagen (Chadstone, Australia) and reverse transcribed to cDNA via SuperScript IV transcriptase kit (Thermo Fisher Aust Pty Ltd, Scoresby, Australia). Real-time PCR was conducted using the LightCycle 480 system (Roche Diagnostics, Castle Hill, Australia) with SYBR green master mix from Roche (Roche Diagnostics, Castle Hill, Australia). Primers for TH, DAT and COMT were sourced from Sigma Aldrich (Castle Hill, Australia), sequences listed in Table 1. The results were quantitated using hypoxanthine guanine phosphoribosyl transferase (HPRT) as the housekeeping gene to determine relative expression.

**Table 1:** Primer Sequences used for real-time PCR.

Gene Name	Primer sequence 5′ – 3′
TH – Forward	GCTTCAATGACGCCAAGGAC
TH – Reverse	CTGGATGGTGTGAGGGCTGT
DAT – Forward	GGGTTTGGAGTGCTGATTGC
DAT – Reverse	GACGACGAAGCCAGAGGAGA
COMT – Forward	ATCTTCACGGGGTTTCAGTG
COMT – Reverse	GAGCTGCTGGGGACAGTAAG

### Statistical Analysis and Calculations

Previously, it was found that the procognitive effects of low dose amphetamine were dependent on baseline performance and therefore drug response was compared between low and high performance groups^24^. The number of animals used was determined by a power analysis using these previous results. A median split (at 80% accuracy) from baseline performance was used to allocate rats into low (*n* = 8) and high (*n* = 10) performance groups. Repeated measures ANOVA was used to compare groups across signal durations at baseline and after amphetamine. Following this performance groups were compared using two-tailed independent *t*-tests and paired *t*-test were used to compare measures within groups. Bonferroni adjustment for multiple comparisons was used where appropriate and Greenhouse-Geisser correction was applied if there was a violation of sphericity.

A previous study has shown a negative correlation between baseline performance and improvement after low dose amphetamine^4^. Because this finding would be supported by regression to the mean effects, baseline accuracy values were subjected to a normalisation adjustment. The following formula was used^19^:

$$\begin{array}{rcl}Adjusted\,baseline,x^{\prime}  & = & initial\,baseline\,score,x+{(1-retestreliability,{r}_{xx})}^{\ast }\\  &  & (mean\,of\,total\,sample,mu-baseline\,score,x)\end{array}$$

Reliability was calculated by correlating accuracy after vehicle treatment within the Latin square design and after vehicle treatment at the end of the drug schedule (Pearson’s correlation, *r* = 0.817). To assess relative improvement in accuracy, a difference score was calculated by subtracting accuracy after vehicle from accuracy after amphetamine. All data were analysed using SPSS software package (ver.20, SPSS Inc. IL, USA). Significance was set at *p* < 0.05 and all data are presented as mean ± S.E.M., *p < 0.05.

## Electronic supplementary material

Supplementary Info File
