# Supplementary material for: Baseline-dependent effects of amphetamine on attention are associated with striatal dopamine metabolism
Source: Sci Rep. 2017 Mar 22;7:297. doi: 10.1038/s41598-017-00437-9 (PMC5428442; doi:10.1038/s41598-017-00437-9)
Supplement: Supplementary file 1 — Supplementary Info File [file 41598_2017_437_MOESM1_ESM.pdf]

Baseline-dependent effects of amphetamine on attention are associated with striatal dopamine metabolism

Karly M. Turner[1], BSc (Hons), BA, PhD
James Peak[1], BSc (Hons)
Thomas H. J. Burne[1,2]* BRurSc (Hons), PhD

1. Queensland Brain Institute, The University of Queensland, St Lucia, QLD 4072, Australia
2. Queensland Centre for Mental Health Research, The Park Centre for Mental Health, Richlands, QLD 4077, Australia

***Address for correspondence**
Assoc. Prof. Thomas Burne
Queensland Brain Institute
The University of Queensland,
St Lucia, Qld 4072
Australia.
Email: t.burne@uq.edu.au
Fax: +61 7 3346 6301
Phone: + 61 7 3346 6371

Supplementary Files:

Supplementary Table 1: Relative ratios of selected monoamines in the PFC and CPU of low and high performing rats

Supplementary Figure 1: Catecholamine levels in the prefrontal cortex (PFC) of low and high performing rats

Supplementary Table 1. Relative ratios of selected monoamines in the PFC and CPU of low and high performing rats

	PFC		CPU	
	Low Performers	High Performers	Low Performers	High Performers
DOPAC/DA	2.47 +/- 0.33	1.91 +/-0.19	0.68 +/- 0.07	0.72 +/-0.10
HVA/DA	1.55+/- 0.18	1.42 +/-0.18	0.16 +/-0.01	0.15 +/-0.02
5-HIAA/5-HT	8.72 +/- 1.30	6.71 +/- 0.73	7.12 +/- 0.54	7.19 +/- 0.52

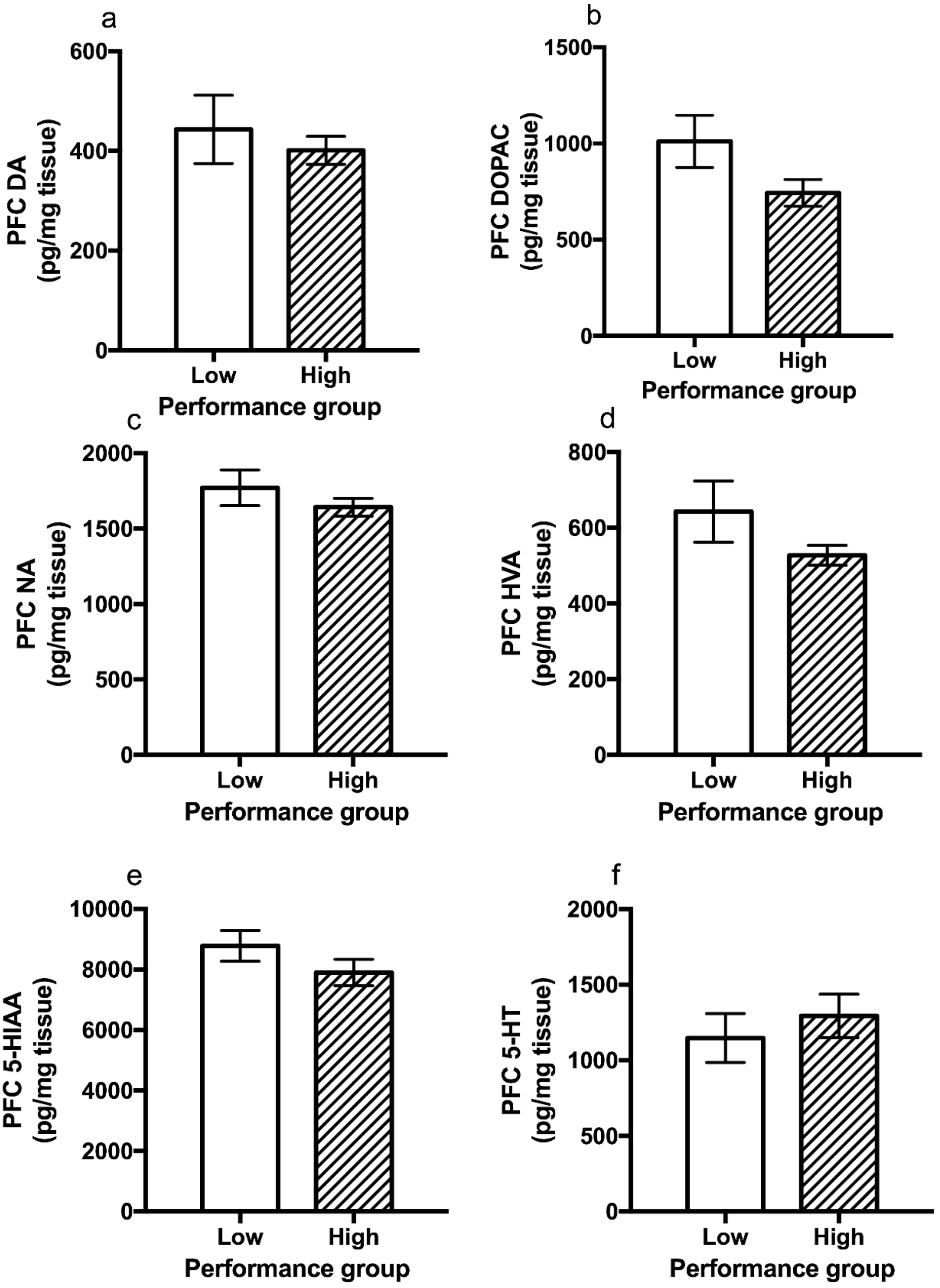

Supplementary Figure 1. Catecholamine levels in the prefrontal cortex (PFC) of low and high performing rats. There were no significant differences between performance groups for (*a*) dopamine (DA), (*b*) dihydroxyphenyl acetic acid (DOPAC) or (*c*) noradrenaline (NA), (*d*) homovanillic acid (HVA), (*e*) 5-hydroxyindoleacetic acid (5-HIAA) or (*f*) serotonin (5-HT).
